# Traces of SARS-CoV-2 RNA in Peripheral Blood Cells of Patients with COVID-19

**DOI:** 10.1089/omi.2021.0068

**Published:** 2021-08-04

**Authors:** Ahmed Moustafa, Rana Salah Khalel, Ramy K. Aziz

**Affiliations:** ^1^ Department of Biology, American University in Cairo, New Cairo, Egypt.; ^2^ Biotechnology Graduate Program, American University in Cairo, New Cairo, Egypt.; ^3^ Bioinformatics and Integrative Genomics Lab, American University in Cairo, New Cairo, Egypt.; ^4^ Department of Microbiology and Immunology, Faculty of Pharmacy, Cairo University, Cairo, Egypt.; ^5^ Center for Genome and Microbiome Research, Faculty of Pharmacy, Cairo University, Cairo, Egypt.; ^6^ Microbiology and Immunology Research Program, Children's Cancer Hospital Egypt 57357, Cairo, Egypt.

**Keywords:** SARS-CoV-2, coronavirus, COVID-19, immunology, PBMC, transcriptomics

## Abstract

The severe acute respiratory syndrome coronavirus 2 (SARS-CoV-2) is the third virus that caused coronavirus-related outbreaks over the past 20 years. The outbreak was first reported in December 2019 in Wuhan, China, but rapidly progressed into a pandemic of an unprecedented scale since the 1918 flu pandemic. Besides respiratory complications in patients with COVID-19, clinical characterization of severe infection cases showed several other comorbidities, including multiple organ failure, and septic shock. To better understand the systemic pathogenesis of COVID-19, we interrogated the virus's presence in the peripheral blood cells, which might provide a form of trafficking or hiding to the virus. By analyzing >2 billion sequence reads of high-throughput transcriptome sequence data from 180 samples of patients with active SARS-CoV-2 infection or healthy controls collected from 6 studies, we found evidence of traces of SARS-CoV-2 RNA in peripheral blood mononuclear cells in two samples from two independent studies. In contrast, the viral RNA was abundant in bronchoalveolar lavage specimens from the same patients. We also devised a “viral spike-to-actin” RNA normalization as a metric to compare across various samples and minimize errors caused by intersample variability in total human RNA abundance. Our observation suggests immune presentation and discounts the possibility of extensive viral infection of lymphocytes or monocytes.

## Introduction

The severe acute respiratory syndrome coronavirus 2 (SARS-CoV-2) is the third virus that caused coronavirus-related outbreaks over the past 20 years. The first outbreak occurred in Asia in 2002–2003, causing SARS; hence, the name SARS-CoV, which back then was not related to any of the known viruses (Marra et al., 2003; Rota et al., 2003). Between 2002 and 2003, 8098 people became sick with SARS, and of those, 774 died (i.e., a mortality rate of 9.5%). Since 2004, there have been no more reports of SARS cases (via NHS, WHO, and CDC).

The second coronavirus-related outbreak started in the Arabian Peninsula in 2012 (Zaki et al., 2012), causing a fatal disease, Middle East respiratory syndrome (MERS), with a significantly higher mortality rate of 40% of the cases infected by MERS-CoV virus (Zumla et al., 2015).

More recently, in December 2019, the third coronavirus-related outbreak was first reported in Wuhan, China, by the emergence of the SARS-CoV-2, initially dubbed “the 2019 novel coronavirus” (2019-nCoV). The spread of the virus led to a pandemic of an unprecedented scale since the 1918 flu pandemic.

As of mid-June 2021, >175 million confirmed COVID-19 cases globally, and >3.5 million deaths have been reported by the WHO (WHO Dashboard, continuously updated). Besides the respiratory complications in patients with COVID-19, clinical characterization of severe infection cases indicated further comorbidities, including multiple organ failure (liver, kidney, and heart) and septic shock (Cascella et al., 2020; Li et al., 2020; Poston et al., 2020).

Since the first genome sequence of SARS-COV-2 has been determined and made public in January 2020 (Lu et al., 2020), >2 million genomes have been sequenced worldwide and become available through the Global Initiative on Sharing All Influenza Data (Shu and McCauley, 2017). The availability of those genomic sequences allows rapid screening of viral RNA in human tissues and environmental samples [e.g., sewage (Bibby and Peccia, 2013)] using multi-omic wet lab technologies and *in silico* screening tools for publicly available metatranscriptomic samples.

To better understand COVID-19 systemic pathogenesis, we conducted this study to interrogate the presence of the virus in the blood, or any of its components, as it might provide a form of trafficking or hiding to the virus, notably that some precarious studies reported the ability of the virus to infect lymphocytes (Wang et al., 2020b). In contrast, others have suggested that the virus exerts its pathogenesis through “attacking hemoglobin,” although this hypothesis has been heavily criticized (Read, 2020). The virus was sporadically reported to be found in the plasma or blood of patients with COVID-19 (Huang et al., 2020; Wang et al., 2020a). Finally, peripheral blood mononuclear cells (PBMCs) were shown to harbor other infectious viruses, such as HIV, HCV, and HBV (Li et al., 2015; Wang et al., 2002).

We computationally analyzed high-throughput sequence data from patients with active COVID-19 in several publicly available RNA-Seq datasets for the reasons mentioned previously. We found only evidence of traces of SARS-CoV-2 RNA in their PMBCs, whereas their bronchoalveolar lavage samples had large amounts of viral RNA.

## Materials and Methods

### Datasets and quality control

Publicly available raw RNA-Seq FASTQ sequences, published by six studies (Arunachalam et al., 2020; Kusnadi et al., 2021; Manne et al., 2020; Wilk et al., 2020; Xiong et al., 2020; Zheng et al., 2020), were retrieved (Table 1). For quality control of raw sequences, fastp (Chen et al., 2018) was used to remove adaptor sequences, trim low-quality ends, and remove short reads.

**Table 1.** RNA-Seq Datasets Included in the Study

Bioproject accession	Healthy Controls		Patients with COVID-19			References
	PBMCs	Platelet	BALF	PBMCs	Platelet	
PRJCA002326	3	0	4	3	0	Xiong et al. (2020)
PRJNA633393	6	0	0	7	0	Wilk et al. (2020)^a^
PRJNA634489	0	5	0	0	10	Manne et al. (2020)
PRJNA639275	34	0	0	32	0	Arunachalam et al. (2020)
PRJNA644579	0	0	0	39	0	Kusnadi et al. (2021)
PRJNA662985	0	0	0	37	0	Zheng et al. (2020)

The numbers of the samples are shown by the condition of the subject, healthy controls, or patients with COVID-19, and the source of the blood sample, BALF, PBMCs, or platelet. The number of included samples is 180 (48 healthy controls and 132 COVID-19).

^a^ These 13 datasets included single-cell RNA-Seq.

BALF, bronchoalveolar lavage fluid; PBMCs, peripheral blood mononuclear cells.

### Detection of viral RNA

Filtered FASTQ sequences were aligned to the SARS-CoV-2 reference genome (GenBank accession NC_045512) by the Burrows-Wheeler aligner (Li and Durbin, 2009). Sambamba (Tarasov et al., 2015) was used to filter generated binary alignment map files for mapped sequences with a quality score >40 and alignment score >90. For further verification, identified SARS-CoV-2 matching sequences were manually inspected, and blastn (Altschul et al., 1990) was used to check them against the NCBI nucleotide “nt” database. Blast matches and alignments were visually reviewed. In addition, the matching sequences were annotated by blastx searches against the NCBI “RefSeq protein” database.

### Viral-to-human expression normalization

To estimate viral RNA abundance in a given sample and make a comparison between samples possible, we normalized the number of any positive SARS-CoV-2 matching hits to the total number of reads within that sample. In addition, we used the spike gene, which is highly specific to SARS-CoV-2, to estimate the extent of viral RNA load in a given sample. We normalized the abundance of spike genes to human actin RNA, being a transcript of a housekeeping gene. This normalization generated a “spike-to-actin” ratio that could be used as an accurate metric for viral RNA abundance relative to human RNA because the total number of reads may include nonhuman and nonviral samples.

### Dimension reduction and clustering

For dimension reduction analysis, we used the *plotPCA* function implemented in the DESeq2 package (Love et al., 2014), after applying the *rlog* function, which transforms the transcript count data to the log2 scale and normalizes the count data to the sizes of the libraries.

### Differential gene expression analysis

Filtered sequences, in FASTQ format, were processed in Salmon (Patro et al., 2017) for the quantification of the expressed transcripts against the human reference GENCODE (Frankish et al., 2019) Release 35 (GRCh38.p13). The DESeq2 package (Love et al., 2014) was used for transcript normalization and differential gene expression analysis.

The comparisons performed for the differential expression are (1) healthy controls versus patients with COVID-19 without viral RNA, (2) healthy controls versus patients with COVID-19 with viral RNA, and (3) patients with COVID-19 without viral RNA versus patients with COVID-19 with viral RNA. The cutoff for statistical significance was adjusted *p* < 0.001. Thus, the complete set of the differentially expressed genes (DEGs) is the union of the identified DEGs from the three comparisons. Patterns of differential gene expression were visualized as a heatmap using the R package pheatmap https://cran.r-project.org/package=pheatmap. Further data processing and visualization were performed in R https://www.r-project.org/. Toppgene (Chen et al., 2009) was used for enrichment analysis for differentially expressed transcripts.

## Results

We analyzed 180 RNA-Seq datasets from 6 independent studies and found SARS-CoV-2 viral sequences in only 2 PBMC samples from 2 independent studies (Table 2). Sample CRR119891 (Xiong et al., 2020) had four viral sequence reads (Supplementary Fig. S1A, B), matching SARS-CoV-2's polyprotein pp1ab (accession NP_828849), with blastx *e*-value 1 × 10^−29^, and surface glycoprotein (accession YP_009724390), with blastx *e*-value 2 × 10^−25^. In addition, sample SRR12626644 (Zheng et al., 2020) had two viral sequence reads (one paired-end read), matching SARS-CoV-2's ORF1a polyprotein (accession YP_009725295), with blastx *e*-value 9 × 10^−53^ (Supplementary Fig. S1C).

**Table 2.** Sample Types and Identified Viral Reads

COVID-19 cell type	No. of samples with viral RNA	Median abundance of viral RNA
Platelets	0	0
PBMCs	2	3.46E-08
BALF	4	1.07E-02

The abundance of viral RNA is estimated as the number of detected viral RNA reads to the total number of RNA reads in the sample.

On the contrary, expectedly, we found viral sequences in all the bronchoalveolar lavage fluid (BALF) samples (Xiong et al., 2020) with a median abundance of 1.07% of the total sequence reads, which, despite being a relatively low percentage of the total reads, was sufficient to cover the entire genome of SARS-CoV-2 with coverage exceeding 4000 × for some areas in the viral genome (Supplementary Fig. S2).

**FIG. 1.**
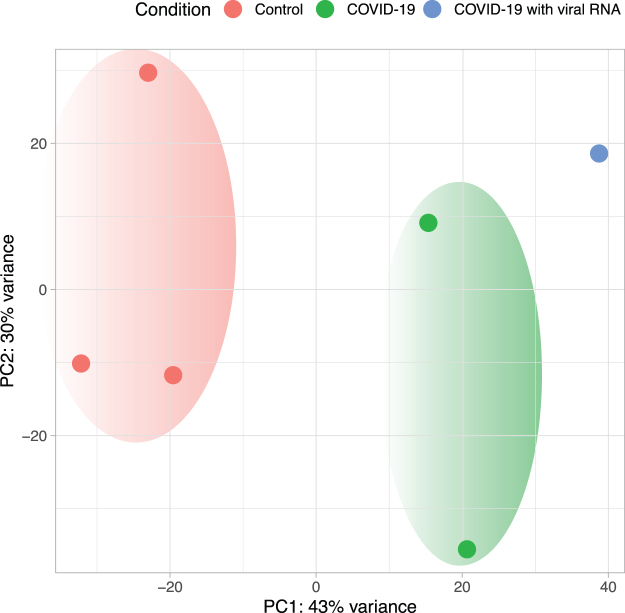
Dimensionality reduction of PBMC transcriptomes. PCA was used to cluster the PBMC samples based on the normalized gene expression. PBMC samples are color-coded: *red,* healthy controls; *green,* patients with COVID-19, but without viral RNA in PBMCs; *blue,* patients with COVID-19 with viral RNA detected in PBMCs. PBMC, peripheral blood mononuclear cell; PCA, principal component analysis.

Based on the normalized gene expression values of the PBMC samples (Xiong et al., 2020), principal component analysis (PCA) demonstrated separation between the COVID-19 and healthy control samples. Moreover, the t-SNE analysis showed a further separation between the patient samples in which viral RNA was detected in PBMCs and those with no detected viral RNA (Fig. 1).

**FIG. 2.**
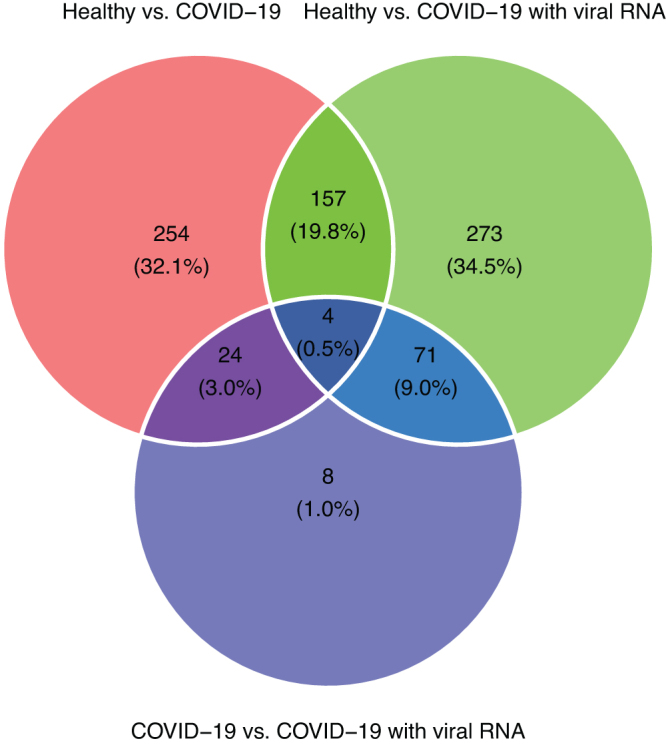
Venn diagram of the counts of DEGs. The numbers are counts of the DEGs and the percentages relative to the total (union) number of DEGs. DEGs, differentially expressed genes.

Our differential gene expression analysis of the PBMC samples (human transcriptome) identified 439 DEGs between the healthy controls and patients with COVID-19 without SARS-CoV-2 viral RNA (adjusted *p* < 0.001), 505 DEGs between healthy controls and patients with COVID-19 with detected SARS-CoV-2 viral RNA (adjusted *p* < 0.001), and 107 DEGs between patients with COVID-19 without detected SARS-CoV-2 viral RNA and patients with COVID-19 with detected SARS-CoV-2 viral RNA (adjusted *p* < 0.001). The pairwise comparisons of the three types resulted in 791 DEGs (Fig. 2).

**FIG. 3.**
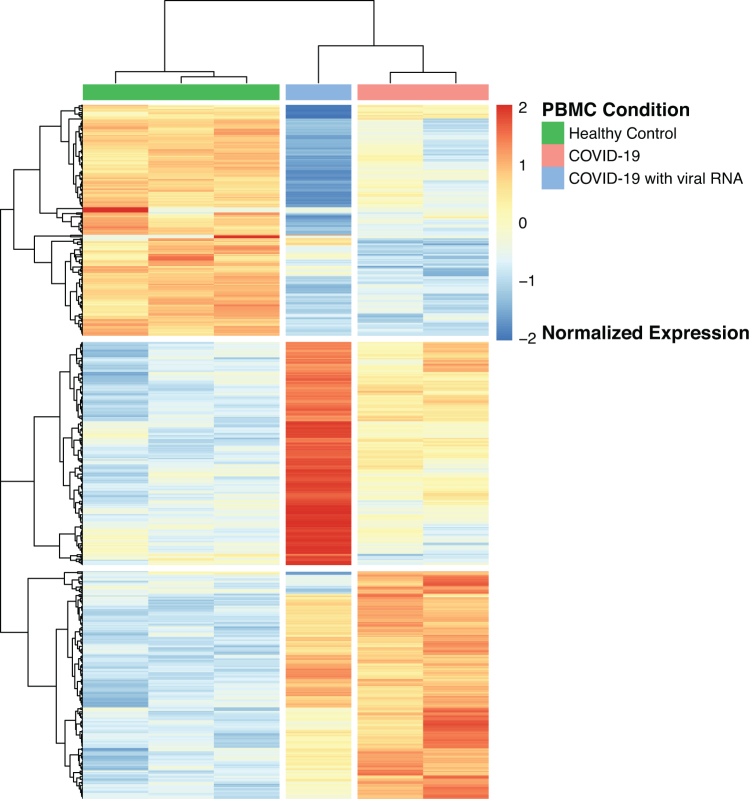
Heatmap of the expression patterns of the DEGs. Control: health control, COVID19: COVID-19 with no detected viral RNA, COVID-19 with detected viral RNA.

In agreement with the PCA analysis, hierarchical clustering delineated the patterns of DEGs and confirmed the separation of the three groups: controls, COVID-19 without SARS-CoV-2 viral RNA, and COVID-19 with SARS-CoV-2 viral RNA (Fig. 3).

Toppgene enrichment analysis for genes that were differentially expressed between healthy controls and COVID-19 samples with viral RNA and between COVID-19 without viral RNA and COVID-19 with viral RNA using provided enriched Gene Ontology (GO) terms under the Molecular Function, Biological Process, and Cellular Component categories (Table 3).

**Table 3.** Statistically Enriched Gene Ontology Terms

GO category	GO term	Description	Adjusted* p*-value
GO: Molecular Function	GO:0003823	Antigen binding	2.53E-02
GO: Biological Process	GO:0051707	Response to other organisms	7.63E-05
GO: Biological Process	GO:0043207	Response to external biotic stimulus	7.96E-05
GO: Biological Process	GO:0009607	Response to biotic stimulus	1.77E-04
GO: Biological Process	GO:0002443	Leukocyte mediated immunity	2.25E-04
GO: Biological Process	GO:0002250	Adaptive immune response	2.55E-04
GO: Biological Process	GO:0045087	Innate immune response	3.41E-04
GO: Biological Process	GO:0002252	Immune effector process	4.77E-04
GO: Biological Process	GO:0002449	Lymphocyte-mediated immunity	6.95E-04
GO: Biological Process	GO:0006958	Complement activation, classical pathway	1.93E-03
GO: Biological Process	GO:0033197	Response to vitamin E	3.19E-03
GO: Biological Process	GO:0016050	Vesicle organization	4.63E-03
GO: Biological Process	GO:0098542	Defense response to other organisms	4.82E-03
GO: Biological Process	GO:0002460	Adaptive immune response based on somatic recombination of immune receptors built from immunoglobulin superfamily domains	5.06E-03
GO: Biological Process	GO:0002455	Humoral immune response mediated by circulating immunoglobulin	6.08E-03
GO: Biological Process	GO:0072376	Protein activation cascade	6.53E-03
GO: Biological Process	GO:0006956	Complement activation	7.48E-03
GO: Biological Process	GO:0019058	Viral life cycle	8.37E-03
GO: Biological Process	GO:0030449	Regulation of complement activation	1.07E-02
GO: Biological Process	GO:0006952	Defense response	1.31E-02
GO: Cellular Component	GO:0019814	Immunoglobulin complex	2.18E-04

Statistically significant enriched GO terms with Bonferroni-adjusted *p* < 0.05.

GO, Gene Ontology.

When the statistically enriched terms were filtered to adjusted *p* < 0.05, the following categories stood out. Under the *Molecular Function* category, there was only one statistically significant enriched term, GO:0003823 (antigen binding). Likewise, only one statistically significant enriched term under the *Cellular Component* category satisfied the statistical filter, that is GO:0019814 (immunoglobulin complex). Under the *Biological Process* category, 19 statistically significant enriched terms were shortlisted. Many of these terms were related to immune responses and viral life cycle, including GO:0051707 (response to other organisms), GO:0002250 (adaptive immune response), GO:0045087 (innate immune response), and GO:0002449 (lymphocyte-mediated immunity).

**FIG. 4.**
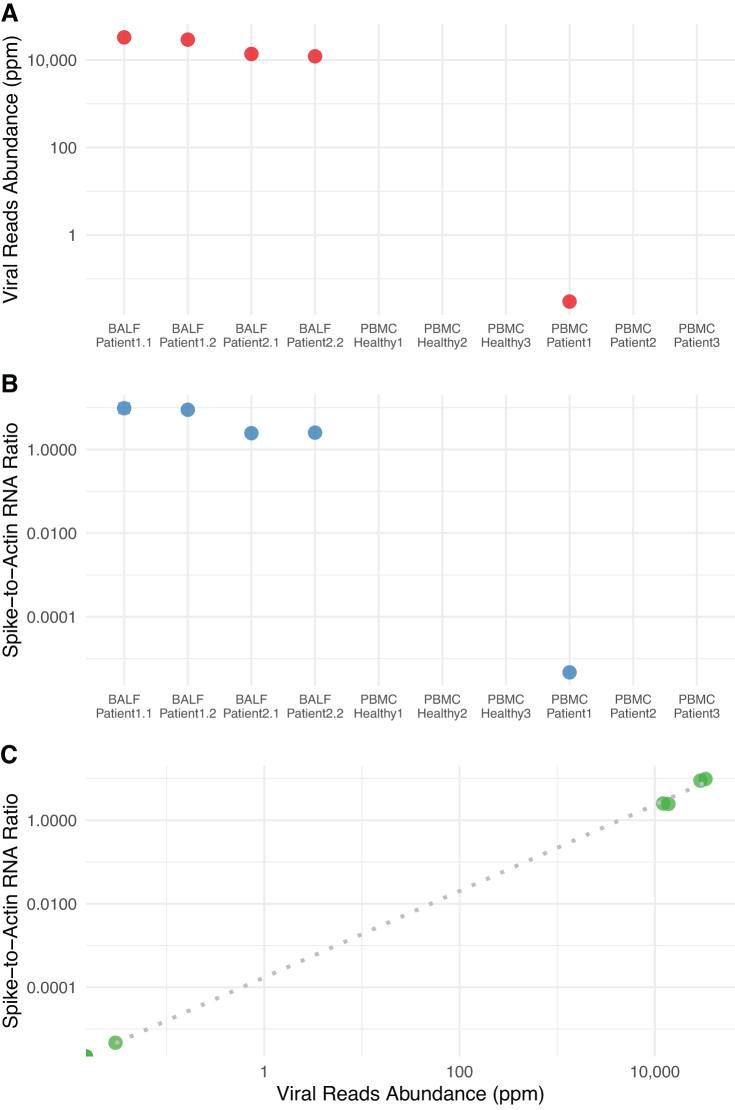
Abundance of SARS-CoV-2 sequence reads relative to the host gene expression. **(A)** Normalized abundance of SARS-CoV-2 sequence reads, estimated as the number of SARS-CoV-2-specific hits per million sequence reads. **(B)** SARS-CoV-2 spike-to-human actin ratio for each sample. **(C)** A scatter plot showing the correlation between SARS-CoV-2 spike-to-human actin ratio and SARS-CoV-2 sequence reads abundance (in ppm). ppm, parts per million; SARS-CoV-2, severe acute respiratory syndrome coronavirus 2.

When we calculated a spike-to-actin RNA ratio for each sample, values for all four BALF samples, as well as the one PBMC sample with SARS-CoV-2 transcripts, followed the same pattern of viral RNA abundance ratios and were strongly correlated (Spearman correlation *ρ* = 0.96, *p* = 0.00047; Fig. 4C)

## Discussion

Coronavirus-related infections are reported to be associated with hematological changes, including lymphopenia, thrombocytopenia, and leukopenia, by infecting blood cells, bone marrow stromal cells, or inducing autoantibodies (Yang et al., 2003). In a former study characterizing the clinical features of patients with COVID-19, Huang et al. (2020) showed that using reverse-transcription polymerase chain reaction (RT-PCR) allowed them to detect coronavirus in plasma-isolated samples from the patients. Their report preferred to use the term “RNAaemia,” rather than “viraemia,” which they defined as the presence of viral RNA in the blood because they did not perform tests to confirm the presence of infectious SARS-CoV-2 virions in the blood of the patients.

A handful of early reports detected amplifiable viral RNA in the blood (Peng et al., 2020; Wang et al., 2020a); however, to our knowledge, no studies used an unbiased systematic approach to report SARS-CoV2 RNA in PBMCs (Azghandi and Kerachian, 2020). In their preliminary analysis of RNA isolated from PBMCs, Corley et al. confirmed that they did not detect viral sequences (a preprint 10.1101/2020.04.13.039263v1). High-throughput sequencing has been repeatedly demonstrated to be a practical approach for the identification and quantification of viruses in the blood (Moustafa et al., 2017) following similar methods as those used in viral metagenomics (Aziz et al., 2015; Breitbart et al., 2003) and uncultivated viral genomics (Roux et al., 2019).

Therefore, we planned to exhaustively mine publicly available RNA-Seq PBMC datasets for SARS-CoV-2 RNA sequences. As of the writing of this report, only one study reported viral RNA in an RNA-Seq PBMC dataset (Xiong et al., 2020). In that study, the authors profiled global gene expression in BALF and PBMC specimens of patients with COVID-19. Predictably, we detected viral RNA in all BALF samples (2 patients, 2 replicates each) with an average abundance of 1.07% of the total RNA reads in those samples, including human RNA (Table 1). We also confirmed the finding by Xiong et al. (2020) and detected the viral RNA sequences in one of their PBMC samples from patients with COVID-19 (Table 1). Furthermore, we identified another PBMC sample in a patient with COVID-19 in a dataset published by Zheng et al. (2020) (Table 1). Those authors described the whole-transcriptome expression profiles in PBMCs of 18 patients with COVID-19 over three time points. However, the authors have not investigated or reported the detection of viral RNA in their samples (Zheng et al., 2020).

Of note, to estimate the viral RNA load in a given sample and to compare between different samples, we normalized viral read counts to the total number of reads in each dataset (Fig. 4). Because the total number of human-related reads may be strongly affected by potential microbial RNA (from pulmonary microbiota, for example) or by extensive viral counts, we also sought to estimate the ratio of viral genomic RNA to human cellular RNA. For that purpose, we used actin RNA, being a transcript for a human housekeeping gene—not expected to be seen in bacterial cells or viral genomes and expected to have constitutive expression levels. We estimated the ratio of hits to RNA encoding the spike protein (S) to actin transcripts in the sample. Quite interestingly, the spike-to-actin ratio strongly correlated with viral RNA abundance values (computed as SARS-CoV-2 RNA reads normalized to the total number of reads) (Fig. 4C).

These RNA traces are indeed quite rare; however, they confidently and unambiguously belong to SARS-COV-2 (Supplementary Fig. S1). One viral RNA read translates into polyprotein (pp1ab, accession NP_828849), the largest protein of coronaviruses and involved in replicating and transcribing the viral genome. The other viral RNA from the same sample translates into surface (spike) glycoprotein (accession YP_009724390), which mediates the entry of the viral RNA into human cells expressing human angiotensin-converting enzyme 2(hACE2) (Ou et al., 2020; Walls et al., 2020). The third viral RNA was from a different sample, and it translated into ORF1a polyprotein (accession number YP_009725295), a leader protein, from which the nonstructural proteins are derived.

In our differential expression analysis of the human transcriptome, we opted to limit the analysis to the samples from the Xiong et al. (2020) study because (1) it includes PBMCs from healthy donors and patients with COVID-19 with and without RNA viral sequences and (2) to exclude possible batch effects across RNA-Seq datasets generated from different studies. Our analysis shows a clear separation between the PBMC transcriptomes from healthy donors and patients with COVID-19 (Figs. 1 and 3). The analysis also shows a further separation within the COVID-19 PBMC samples between samples without detected viral RNA and the sample with viral RNA, suggesting that the PBMC transcriptome with the presence of the viral RNA differs from that with no detected viral RNA sequences.

Although we do not reject the possibility of cross-contamination or barcode bleeding (Kircher et al., 2012; Mitra et al., 2015) for detecting the viral RNA in two PBMC RNA-Seq samples, such possibility is unlikely, given that control samples had no hits to SARS-CoV-2 RNA. A more likely possibility is that SARS-CoV-2 is being sampled by antigen-presenting cells (most likely dendritic cells) or presented to T lymphocytes, which are in the PBMC population. Because such an event (antigen presentation) may be relatively difficult to detect, this explains why it was detected in only 2 of 118 datasets.

One more possibility, which needs many more samples to consider, is that SARS-CoV-2 may be transiently or coincidentally internalized by one of the mononuclear cell types, suggesting a mechanism for the chronicity of the SARS-CoV-2 infection. This hypothesis requires further testing. However, in light of our analysis, it is difficult to support the early reports that SARS-COV-2 targets T lymphocytes *in vivo*, as suggested earlier, in a correspondence, based on cell culture experiment with pseudotyped viruses (Wang et al., 2020b).

It is important to emphasize that, after 1.5 years of the start of the COVID-19 pandemic, a scientific consensus has been reached that this disease is not merely respiratory, but is a systemic disease involving multiple organs. Our results about the paucity of detectable SARS-CoV-2 RNA in peripheral blood cells are not contradictory to the systemic nature of the disease or the possibility of viral transport through the circulatory system. What the study reports, quite specifically, is that PBMCs are not specifically targeted by the virus. This finding is supported by reports on the lack of ACE2 expression by those immune cells (Hamming et al., 2004; Salamanna et al., 2020).

Although publicly available SARS-CoV-2 genomic data have accumulated to a historical record (>2 million genome sequences), blood transcriptome and RNA-seq data remain insufficient. With more data becoming publicly available, it will be possible to revisit this hypothesis and others to improve our understanding of the progression, replication, chronicity, and recurrence of SARS-CoV-2 in infected individuals.

## Supplementary Material

Supplemental data

Supplemental data

## Abbreviations Used

AUC: American University in Cairo
BALF: bronchoalveolar lavage fluid
DEGs: differentially expressed genes
GO: Gene Ontology
hACE2: human angiotensin-converting enzyme 2
MERS: Middle East respiratory syndrome
PBMCs: peripheral blood mononuclear cells
PCA: principal component analysis
SARS-CoV-2: severe acute respiratory syndrome coronavirus 2
