# Supplementary material for: Traces of SARS-CoV-2 RNA in Peripheral Blood Cells of Patients with COVID-19
Source: OMICS. 2021 Aug 4;25(8):475–83. doi: 10.1089/omi.2021.0068 (PMC8377512; doi:10.1089/omi.2021.0068)

**Figure S1. Alignment of the detected viral RNA sequences to the SARS-CoV-2 genome.** A pairwise alignment between the SARS-CoV-2 reference genome (GenBank accession [‎NC_045512](https://www.ncbi.nlm.nih.gov/nucleotide/NC_045512)) and the detected viral RNA reads in the analyzed RNA-Seq samples. A perfect match between the reference and the two overlapping mates is indicated by asterisks (“*”). **A.** Sample CRR119891’s read V300046811L2C002R0690997317, which belongs to the region of ORF1ab polyprotein. **B.** Sample CRR119891’s read V300046811L2C004R0360826943, which belongs to the region of surface glycoprotein. **C.** Sample SRR12626644’s read SRR12626644.2111414, which belongs to the region of ORF1a polyprotein.


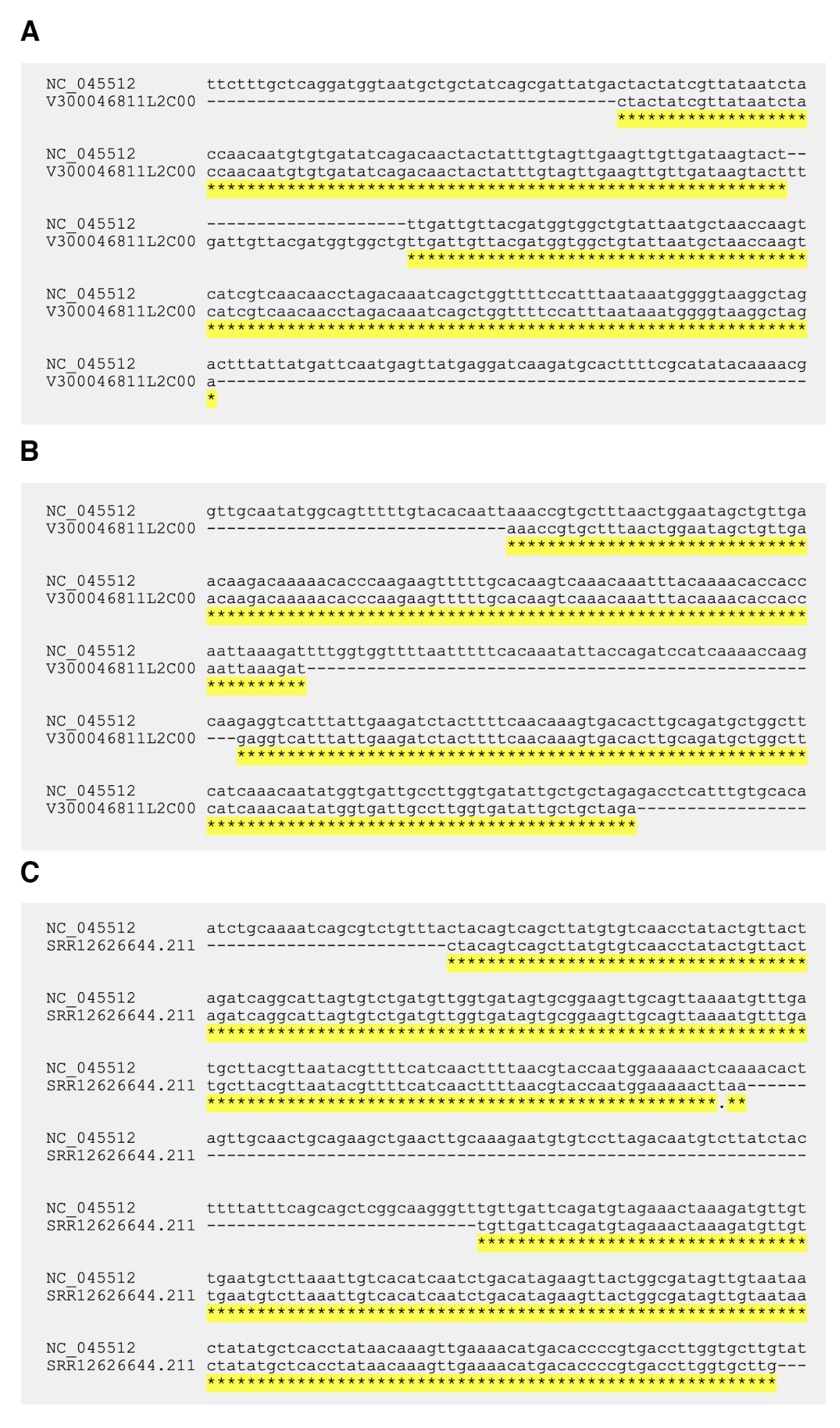

Supplement: Supplemental data [file Supp_Fig1.docx]
