# Supplementary material for: Traces of SARS-CoV-2 RNA in Peripheral Blood Cells of Patients with COVID-19
Source: OMICS. 2021 Aug 4;25(8):475–83. doi: 10.1089/omi.2021.0068 (PMC8377512; doi:10.1089/omi.2021.0068)

**Figure S2. Mapping of SARS-CoV-2 RNA against the SARS-CoV-2 genome.** Viral RNA sequences from BALF samples from patients with COVID-19 are mapped against the SARS-CoV-2 reference genome (GenBank accession [‎NC_045512](https://www.ncbi.nlm.nih.gov/nucleotide/NC_045512)). The x-axis is the nucleotide position on the viral genome. The y-axis is the coverage (in reads; not normalized) of the genomic position by RNA-Seq viral sequences.


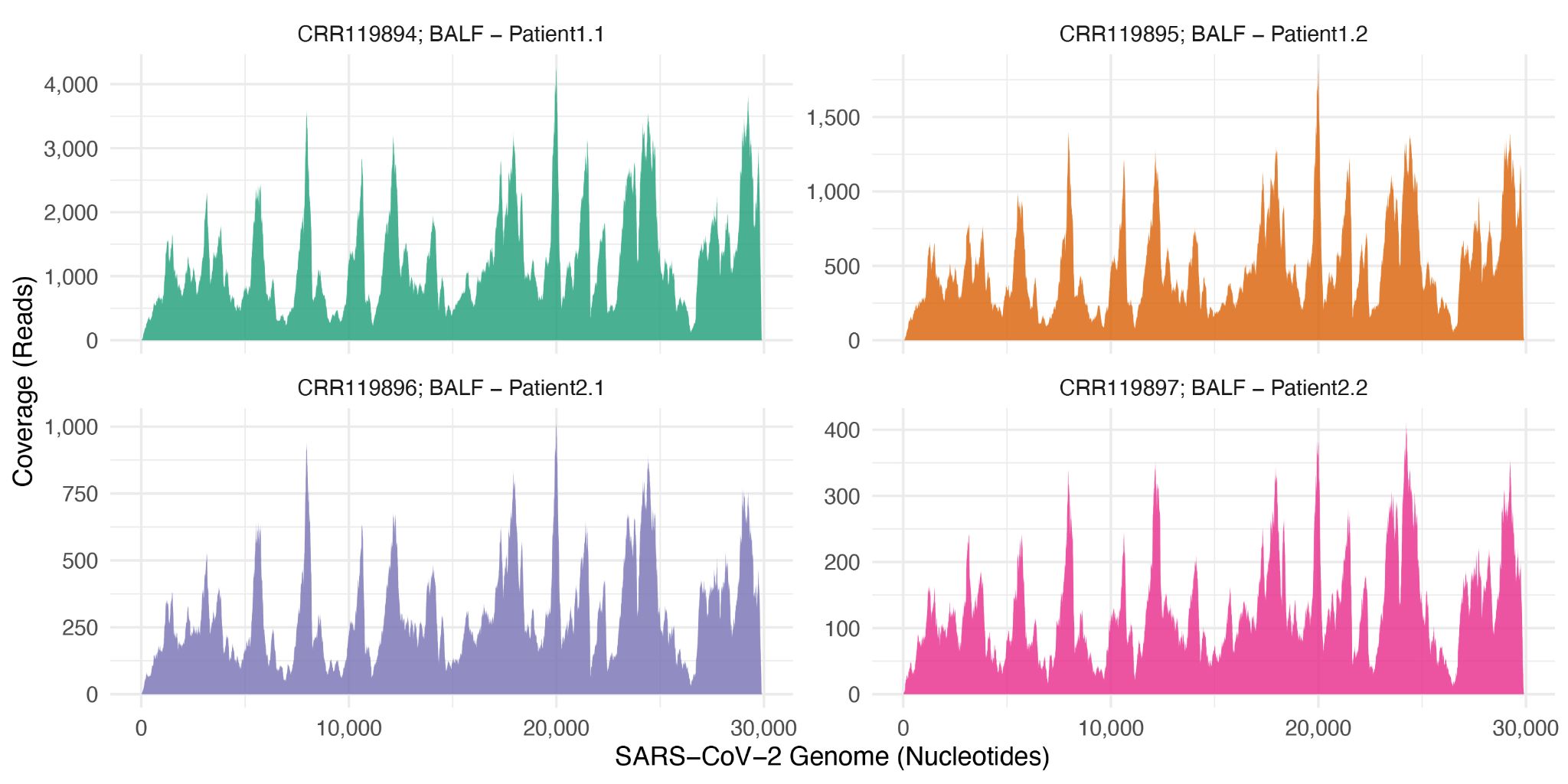

Supplement: Supplemental data [file Supp_Fig2.docx]
